# GBA Regulates EMT/MET and Chemoresistance in Squamous Cell Carcinoma Cells by Modulating the Cellular Glycosphingolipid Profile

**DOI:** 10.3390/cells12141886

**Published:** 2023-07-18

**Authors:** Laura E. Clark, Amanda J. G. Dickinson, Santiago Lima

**Affiliations:** 1 Department of Biology, Virginia Commonwealth University, Richmond, VA 23284, USA; clarkle4@vcu.edu (L.E.C.); ajdickinson@vcu.edu (A.J.G.D.); 2 Massey Cancer Center, Richmond, VA 23298, USA

**Keywords:** glucosylceramide, gangliosides, squamous cell carcinoma, chemoresistance, EMT, MET, sphingolipid, glycosphingolipid

## Abstract

Glycosphingolipids (GSL) are plasma membrane components that influence molecular processes involved in cancer initiation, progression, and therapeutic responses. They also modulate receptor tyrosine kinases involved in EMT. Therefore, understanding the mechanisms that regulate GSLs in cancer has important therapeutic potential. One critical regulator of GSLs is the lysosomal glucosylceramidase β1 (GBA) that catalyzes the last step in GSL degradation. We show that, in cancer, GBA copy number amplifications and increased expression are widespread. We show that depleting GBA in squamous cell carcinoma cell lines results in a mesenchymal-to-epithelial shift, decreased invasion and migration, increased chemotherapeutic sensitivity, and decreased activation of receptor tyrosine kinases that are involved in regulating EMT. Untargeted lipidomics shows that GBA depletion had significant effects on sphingolipids and GSLs, suggesting that increased GBA activity in cancer sustains EMT and chemoresistance by modulating receptor tyrosine kinase activity and signaling via effects on the cellular lipid profile.

## 1. Introduction

Glycosphingolipids (GSLs) are structural components of the outer leaflet of the plasma membrane, where they have many biologically important functions in normal cells. In cancer, alterations in GSL levels have been shown to influence molecular processes involved in disease initiation, progression, and responses to therapy [1]. Among cellular mechanisms, GSLs can modulate receptor tyrosine kinase (RTK) activity and signaling [2,3] and influence the epithelial-to-mesenchymal transition (EMT) [4,5,6]. In addition, the precursor for most cellular GSLs, glucosylceramide (GlcCer), is strongly associated with intrinsic and acquired multidrug resistance in cancer cell lines and in the tumors of patients that failed to respond to chemotherapy [7]. Many cancers have altered GSLs, and our group and others have shown that, compared to normal adjacent uninvolved tissues, GlcCer and lactosylceramide are significantly elevated in tumors [8,9,10]. Therefore, because GlcCers are central players in the metabolism of GSLs, it is important to understand how their levels are regulated and what roles they play in transformed cells.

Most GSLs are synthesized from GlcCer in the Golgi and are exported to the plasma membrane. They re-enter the cytosolic space via endosomes [11], and they are sequentially degraded in the endo-lysosomal compartment. Their basic components such as ceramide or sphingosine are then recycled via the sphingolipid salvage pathway. In lysosomes, the last step in GSL degradation is catalyzed by a single enzyme, the lysosomal glucosylceramidase β1 (GBA), which hydrolyzes the β-glycosidic bond of GlcCer to produce ceramide and glucose. Thus, GBA is a critical regulator of the cellular pool of GlcCer, which is a key player in the etiology and therapeutic responses of human cancers [12,13].

As evidence of the critical role that GBA plays in maintaining cellular homeostasis, mutations in GBA that decrease its catalytic activity and lead to the accumulation of GlcCer and other lipids can lead to the development of debilitating and deadly diseases. Individuals carrying GBA mutations that decrease its activity develop characteristic “Gaucher cells”, which can lead to the development of dementia, hepatomegaly, splenomegaly, Parkinsonism, and peripheral neuropathies [14]. In infants, this may lead to death before the age of 5 [14]. Importantly, although GBA only degrades GlcCer, cells harboring GBA deficiencies or inactivating mutations accumulate other GSLs including gangliosides, sphingosine, ceramides, sphingomyelins, phospholipids, and cholesterol [15,16,17]. Gaucher cells also have altered plasma membrane properties and altered RTK signaling [18]. In addition, somatic mutations in GBA that reduce its activity have also been associated with Parkinson’s disease and the formation of Lewy bodies, with patients suffering from the disease being five times more likely to carry GBA mutations [19]. These are also considered one of the most significant risk factors for Parkinson’s disease [20]. Therefore, the effects of loss of GBA activity are not constrained uniquely to GlcCer accumulation, but reach far beyond and result in significant changes in the cellular lipid profile and membrane properties [21].

Results from recent studies have also highlighted a potentially important role for GBA in cancer [22,23,24]. In ovarian cancer, it was shown that GBA expression is upregulated, and depletion with siRNA resulted in increased sensitivity to chemotherapy, as well as a reduction in signaling via the RTK Axl pathway [24]. In another study, it was shown that GBA expression increased in the tumors of patients with gastric cancer following chemotherapy; moreover, in gastric cancer cell lines, GBA expression was strongly correlated with chemoresistance, growth, and survival [23]. It has also been shown that in hepatocellular carcinoma-derived cell lines reducing the expression of GBA led to increased malignancy, metastasis, and an EMT shift [22]. However, a mechanism that explains why alterations in GBA expression led to these changes was not proposed. In this report, we use molecular biology approaches and mass spectrometry analysis to examine the effects of GBA deletion in two cancer cell lines, and we identify a potential mechanism to explain why GBA alterations in cancer impact malignancy.

We previously showed that GlcCer of various acyl chain lengths are significantly increased in the tumors of patients with lung adenocarcinoma, lung squamous cell carcinoma (SCC), head and neck SCC, endometroid endometrial carcinoma, and colorectal adenocarcinoma [8,9,10]. Yet, as we show in this report, GBA copy number amplifications and increased protein and mRNA expression are common features in tumors of these and many other human cancers. Therefore, the tumors of patients have significantly higher levels of GlcCer, the substrate of GBA; however, paradoxically, these cancers also have significantly higher GBA expression levels. The exception to this paradoxical observation is hepatocellular carcinoma that has high GBA expression, but we showed that tumors of patients with the disease have significantly lower GlcCer and LacCer [8]. Because of these contrasting cancer-specific effects, it appears that the role of GBA in cancer is not fully understood. Since GBA is a critical regulator of GlcCer and ceramide pools, which have such well-established and important roles in human cancers, further investigation of the role of GBA in transformed cells is warranted.

To further study the role of GBA in cancer, we depleted it using genetic approaches in two SCC tumor-derived cell lines, HeLa and NCI-H1703. GBA deletion resulted in significantly increased levels of GlcCer, increased sensitivity to several clinically relevant chemotherapeutic agents, a mesenchymal-to-epithelial (MET) shift, decreased migration and invasion, and reduced activation of various RTKs involved in the maintenance of EMT. Importantly, using untargeted lipidomics, we observed that GBA depletion resulted in changes in the balance of GSL in the plasma membrane, including of gangliosides GM1 and GM2, which are known to modulate RTK activity [25].

## 2. Materials and Methods

### 2.1. Cell Culture

HeLa (CCL-2) and H1703 (NCI-H1703) cells were purchased from the American Tissue Type Collection. Cells were grown at 37 °C in an incubator under atmosphere containing 5% CO_2_ in air_._ HeLa cells were cultured in Eagle Modified Medium (EMEM, 112-018-101, Quality Biological, Gaithersburg, MD, USA) containing nonessential amino acids, 2 mM L-glutamine, 1 mM sodium pyruvate, and 1500 mg/L sodium bicarbonate, supplemented with 10% fetal bovine serum (FBS, 35-011-CV, Corning, Corning, NY, USA), and grown at 37 °C and 5% CO_2_. H1703 cells were cultured in Roswell Park Memorial Institute (RPMI, Corning 10-040-CV) 1640 containing 2 mM L-glutamine, 10 mM HEPES, 1 mM sodium pyruvate, 4500 mg/L glucose, and 1500 mg/L sodium bicarbonate, supplemented with 10% FBS.

### 2.2. Cell Viability and Proliferation Assays

Cell viability assays were performed in 96-well tissue culture treated plates seeded with 10,000 cells per well in full medium containing 10% FBS. After 24 h, cells were treated with chemotherapeutic agents or a vehicle, and allowed to grow for 72 h. Paclitaxel, vinorelbine, and doxorubicin were dissolved in DMSO. Cisplatin solutions were prepared in water; they were either used immediately after preparation or discarded if stored at 4 °C for longer than 7 days. Cell viability was determined by measuring the 450 nm absorbance of WST-8 formazan [2-(2-methoxy-4-nitrophenyl)-3-(4-nitrophenyl)-5-(2,4-disulfophenyl)-2H-tetrazolium, monosodium salt] (CCK-8; CK04, Dojindo Molecular Technologies, Rockville, MD, USA) on a microplate plate reader (Biotek, Winooski, VT, USA). Proliferation assays were performed by seeding 2500 cells per well of a 96 well plate in full medium. Cells were allowed to attach for 18 h and then cell growth determined every 24 h using the WST-8 assay.

### 2.3. Immunoblotting

Cells were cultured in full medium for 48 h and harvested by aspirating the culture medium, and then washing the cells twice with 5 mL of ice-cold PBS. Cells were then scraped in buffer containing 20 mM HEPES, pH 7.4, 250 mM NaCl, 1% Triton X-100, 20% glycerol, and Halt protease plus phosphatase inhibitors (78440, ThermoFisher Scientific, Waltham, MA, USA). The cell suspensions were then sonicated (SFX250/SFX550, Branson Ultrasonics Sonifier, Brookfield, CT, USA) on ice with a microtip for 5 s. Samples were then centrifuged (15,000× *g*, 10 min, 4 °C), and the supernatants were analyzed with the Bio-Rad Protein Assay kit (5000006, Bio-Rad, Hercules, CA, USA) and normalized for protein concentration. Equal amounts of supernatant proteins were resolved by SDS-PAGE, and then transferred onto 0.2 µm polyvinylidene fluoride membrane (1620177, Bio-Rad, Hercules, CA, USA). Immunoblotting was performed with the following antibodies diluted in TBS (50 mM Tris-Cl, pH 7.5, 150 mM NaCl) containing 0.1% Tween-20 (P9416, MilliporeSigma, Burlington, MA, USA) and 2% bovine serum albumin faction V (MilliporeSigma, 126609): GBA (1:000, MilliporeSigma, HPA006667), GBA (1:1000, 88162, Cell Signaling Technology), GAPDH (1:3000, 2118, Cell Signaling Technology, Danvers, MA, USA), vimentin (1:1000, Cell Signaling Technology, 5741), N-cadherin (1:1000, Cell Signaling Technology, 13116), β-catenin (1:1000, Cell Signaling Technology, 8480), SNAIL (1:1000, Cell Signaling Technology, 3879), SLUG (1:1000, Cell Signaling Technology, 9585), ZEB1 (1:1000, Cell Signaling Technology, 3396), E-cadherin (1:1000, Cell Signaling Technology, 3195), pan-keratin (1:1000, Cell Signaling Technology, 4545), and phospho-Akt-Ser473 (1:1000, Cell Signaling Technology, 9271). Immunoblots were visualized by chemiluminescence produced from horseradish peroxidase-conjugated secondary antibodies (1:5000, anti-rabbit 111035045, anti-mouse 115035166; Jackson Immuno Research Labs, West Grove, PA, USA) diluted in TBS containing 0.1% Tween-20, 5% blotting grade milk (Bio-Rad, 1706404), and Super-Signal West Pico (ThermoFisher Scientific, PI-34078) or Dura (ThermoFisher Scientific, PI-34076) chemiluminescent substrates. Blots were imaged using a ChemiDoc MP Imaging System (Bio-Rad) with exposure times adjusted to produce images within the linear range of the instrument. For quantification, immunopositive bands were analyzed using densitometry with Fiji/ImageJ 1.53 [26], and statistical analysis was performed using Prism 8 (GraphPad, Boston, MA, USA).

### 2.4. Mass Spectroscopy

Cells were seeded at a density of 350,000 cells per well in six-well tissue culture-treated plates and grown for 48 h in full medium containing 10% FBS. Cells were harvested by aspirating the medium, washing twice with ice-cold potassium phosphate saline (PBS, ThermoFisher Scientific), and then scraping in 200 µL of ice-cold PBS supplemented with Halt protease and phosphatase inhibitors (ThermoFisher Scientific, 78440). Typically, 100–150 µL of cell suspension was added to 1 mL of ice-cold LC–MS/MS-grade methanol, and LC–MS/MS-grade chloroform was added to reach a ratio of 2:1:0.1 methanol, chloroform, and water, respectively, before storing at −80 °C until processing. Prior to lipid extraction, 10 µL was added of a solution (ethanol:methanol:water, 7:2:1) containing 250 pmol of C17-sphingosine, (2S,3R,4E)-2-aminoheptadec-4-ene-1,3-diol (d17:1-So), C17-sphingosine 1-phosphate, and heptadecasphing-4-enine-1-phosphate (d17:1-So1P). Standards for N-acyl sphingolipids were the following C12 fatty acid analogs: C12-Cer, N-(dodecanoyl)-sphing-4-enine (d18:1/C12:0), C12-Cer 1-phosphate, N-(dodecanoyl)-sphing-4-enine-1-phosphate (d18:1/C12:0-Cer1P), C12-SM, N-(dodecanoyl)-sphing-4-enine-1-phosphocholine (d18:1/C12:0-SM), and C12-glucosylceramide, N-(dodecanoyl)-1-β-glucosyl-sphing-4-eine [9]. All lipids were from Avanti Polar Lipids (Alabaster, AL, USA). The remainder of the cell suspension was sonicated and centrifuged, and the supernatant was analyzed using a Bradford assay (Bio-Rad) to determine protein concentration. Mass spectrometry data were normalized per milligram of protein input. Sphingolipids were quantified by liquid chromatography with electrospray ionization tandem mass spectrometry (LC–ESI-MS/MS, 5500 QTRAP, ABI) with the addition of retention time standards as previously described [8,9]. All solvents and water were LC–MS-grade.

### 2.5. Untargeted Lipidomic Mass Spectrometry Analysis

Untargeted analysis was performed at the VCU Lipidomics and Metabolomics core. A total of 2.5 × 10^6^ cells were seeded on 100 mm dishes and grown for 48 h in medium containing 10% FBS; the cells were washed 2× with ice-cold PBS and harvested in 400 µL of PBS containing Halt protease and phosphatase inhibitors (Thermo Fisher Scientific, 78440). The cell suspensions were then sonicated (35% power, Branson Ultrasonics Sonifier SFX250/SFX550) on ice with a microtip for two rounds of 5 s sonication, followed by 30 s rest on ice. Cell extract suspensions were kept on ice and the protein concentration was determined using the Bradford protein assay. Cell extracts were then equilibrated to matching protein concentrations by diluting with PBS + Halt as appropriate. A total of three rounds of Bradford protein assay and dilution were typically required to achieve equal protein concentration across all samples. An aliquot (150 µL) of each cell suspension was then added to 1 mL of LC–MS-grade ice-cold methanol and lipids extracted as described above for mass spectrometry analysis, but SPLASH LipidomixTM (Avanti Polar Lipids) was added to samples prior to extraction. After drying, lipids were reconstituted with 150 µL of LC–MS-grade methanol. A pooled QC sample was generated with an equal volume of each sample, and then used to condition the column and monitor the intensity variation of internal standard every 10 samples. Next, 10 µL of the reconstituted samples was injected using a Vanquish ultrahigh-performance liquid chromatography system coupled to a Q-Exactive HF mass spectrometer (Thermo Fisher Scientific) with Accucore Vanquish C18 column (150 mm × 2.1 mm × 1.5 µm; Thermo Fisher Scientific) at 55 °C. The mobile phases were (A) acetonitrile/water (50:50 *v*/*v*) and (B) isopropyl alcohol/acetonitrile/water (88:10: 2 *v*/*v*) containing 5 mM ammonia formate and 0.1% formic acid ammonium in each mobile phase. All solvents were LC–MS-grade. The flow rate was set to 0.26 mL/min. The following gradient was applied for lipid separation: 1% (B) from 0–1.0 min; ramped up to 35% (B) from 1.0–3.0 min; 60% (B) at 9 min; 100% (B) at 20 min; constant for 5 min; dropped to 1% (B) at 25.1 min; kept for 4 min for equilibrium. A heated electrospray ionization source (HESI-II) was used for mass spectrum analysis with the following parameters: sheath gas, 17; auxiliary gas, 8; capillary temperature, 325 °C; S-lens RF level, 50; spray voltage, 3.5 kV. The scan ranged from 300 to 2000 m/z with the resolution of 120,000 at m/z of 200. The automatic gain control target was set at 3.0 × 10^6^ and maximum injection time was 100 ms. The top 10 most abundant peaks from a full MS scan were acquired for data-dependent fragmentation (ddMS2). The resolution for ddMS2 was set at 30,000 with normalized collision energy stepped at 20, 30, and 40 with an automatic gain control target of 1 × 10^5^ and a maximum injection time of 50 ms.

The program of Lipidsearch (ThermoFisher Scientific, Version 4.2.21) was used for peak picking, alignment, and lipid annotation. The following parameters were used to remove false-positive endogenous features: void column time of 1 min, RSD of QCs of 30%, the presence of features of 80%, and six times the ratio of QC intensity to method blanks. The tolerance time was set as 0.4 min for annotation combination. The duplicated annotations from positive and negative mode were removed on the basis of intensity. Data normalization was operated with MSTUS (by dividing each intensity of lipid profile by a total intensity of each mode) and EigenMS (a singular value decomposition method to preserves group differences and removed bias) approaches prior to statistical analysis [27].

### 2.6. GBA Knockout Cells

HeLa and H1703 cells were from ATCC. GBA knockouts were generated using CRISPR/Cas9 using predesigned plasmids targeting the GBA loci (KN201614BN; Origene, Rockville, MD, USA) following the manufacturer’s guidelines. Briefly, 3 × 10^5^ cells were seeded in each well of a six-well dish and allowed to attach. The following day, cells were transfected using PolyJet reagent (SL100688; Signagen, Frederick, MD, USA) mixed with 1 μg of each gRNA and donor DNA vectors targeting the GBA loci. After 72 h, cells were exposed to 700 µg/mL G418 (ThermoFisher Scientific, 10131027) and treated continuously for 96 h with daily medium changes and replenishment of G418. Cells were then allowed to expand for 3 days, and then single cells were isolated into 96-well plates by sorting blue fluorescent protein-positive cells using fluorescence-activated cell sorting at the VCU Flow Cytometry Core. Cells were allowed to recover and grow for 7 days, and then selected and examined for expression of GBA using Western blotting.

### 2.7. Statistical Analyses

Most statistical analyses were performed using Prism 8 (GraphPad). All results are expressed as the means ± standard error of the mean. For Western blot densitometry, statistical analyses were performed using an unpaired two-tailed Student *t*-test for comparison of two groups. For cytotoxicity assays, groups were compared using ANOVA. All experiments were independently repeated at least three times with representative data shown in figures. For all analyses, a *p*-value ≤ 0.05 was considered statistically significant.

Untargeted lipidomic PCA analysis was performed using MetaboAnalyst 5.0 with a post hoc Tukey test and false discovery rate (FDR) correction, and then used to compare the normalized annotated lipids every two groups; adjusted *p*-values of 0.05 and 0.1 were considered significant. Hierarchical clusters were generated with R (version 4.2).

### 2.8. Phospho-RTK Proteome Profiler Analysis

Experiments were performed using the Proteome Profiler Human Phospho-RTK Array Kit (ARY001B; R&D Systems, Minneapolis, MN, USA). Two 10 cm tissue culture plates were seeded with 3 × 10^6^ cells and allowed to grow for 48 h. All experiments were performed in parallel with control and GBA knockout cells. To examine the effect of GBA depletion on RTK activation, cells were grown in FBS-free medium for 3 h at 37 °C with 5% CO_2_; then, FBS was added to a final concentration of 10%, and the cells were incubated for 30 min. Plates were then placed on ice, the supernatant was aspirated, and cells were washed twice with ice-cold PBS and drained. Cells were harvested in 1.5 mL of lysis buffer 17. Lysates were prepared following the manufacturer’s guidelines with the following modifications: cell suspensions were rocked in lysis buffer for 1 h and contained Halt protease and phosphatase inhibitor (ThermoFisher Scientific, 78440). Cell lysates containing a total of 300 µg of protein in 1 mL of extract buffer + 500 uL array buffer were incubated with proteome profiler membranes and incubated overnight at 4 °C with rocking. For detection of immunopositive spots, membranes incubated with extracts from control and GBA knockout cells were simultaneously developed with chemiluminescence reagent and imaged in parallel on a ChemiDoc MP Imaging System (Bio-Rad). Duplicate spots were quantified using densitometry with Fiji/ImageJ 1.53 [26], and statistical analysis was performed using Prism 8 (GraphPad). Experiments were repeated three times.

### 2.9. Migration Assays

A total of 225,000 cells were seeded per well of a 12-well plate in 1 mL of medium containing 10% FBS. Plates were incubated at 37 °C with 5% CO_2_ for 48 h, and then scratched using a sterile 200 µL pipette tip. For quantification purposes, it was important to make scratches whose edges fit entirely within the field of view of the microscope objective. Clean tips were used for each well. After scratching, wells were gently washed with 1 mL of sterile PBS to remove loose cells, and then 2 mL of fresh medium was added to the well. Cell migration was monitored with a BZ-X810 microscope (Keyence, Itasca, IL, USA) equipped for time-lapse imaging and a Hit STR stage top environmental chamber incubator (Tokai, Bala Cynwyd, PA, USA). Migration assays were performed at 37 °C with 5% CO_2._ Images were captured using a 10× lens objective in brightfield mode. For data processing, images were analyzed with ImageJ/Fiji 1.53 using the freehand selection tool to outline the wound edges and measure total wound area. Relative closure was calculated for each time point using the equation (1 − (current wound area)/(initial wound area)) × 100. Statistical analysis was performed with ANOVA (Prism 8).

### 2.10. Invasion Assays

Assays were performed using Matrigel (Corning 356234). All pipette tips, inserts, tubes, and any other materials used to handle Matrigel were prechilled at −20 °C. The day prior to experiments, the Matrigel was thawed by placing the vial on ice and incubating overnight at 4 °C. The next day, the Matrigel was diluted to a final concentration of 0.5 mg/mL in prechilled serum-free medium. Next, 100 µL of the diluted Matrigel was added to the top of each transwell insert (8.0 µm, 12 mm diameter, MilliporeSigma PI8P01250), and then incubated at 37 °C and 5% CO_2_ for 3 h. Inserts containing Matrigel were then placed in a 24-well plate (662160; Grenier Bio-One, Monroe, NC, USA) containing 750 µL of medium and 10% FBS that was pre-equilibrated to 37 °C and 5% CO_2_. Prior to harvesting, cells were pre-starved in serum-free medium for 2 h and trypsinized, with the concentration then adjusted to 500,000 cells/mL in serum-free medium. A total of 100,000 cells in 200 µL of serum-free medium were then added to the top chamber of the transwell inserts containing the solidified Matrigel. Cells were then incubated for 48 h at 37 °C and 5% CO_2_.

After 48 h of growth, the inserts were transferred to a clean 24-well plate, all medium was removed, and the inner and outer inserts were washed twice with PBS and drained, and then transferred to a clean 24 well plate. Invading cells (on the outer face of the transwell insert) were then fixed with 4% paraformaldehyde for 5 min, washed twice with PBS, incubated in 100% methanol for 20 min, and washed again twice with PBS. Migrating cells on the outer face of the transwell were then stained with a 0.2 µm filtered 0.5% crystal violet solution in water, and then washed with PBS until all the crystal violet solution was removed. Cells and Matrigel in the inner chamber of the transwell were then thoroughly but carefully removed using a cotton-tipped swab that had been dampened with PBS. The outer surfaces of transwells containing the migrating cells were then imaged using a stereoscope using a white background. Migrating cells were manually counted for quantification. *t*-Tests (Prism) were used to test for statistical significance.

### 2.11. Immunofluorescence Staining and Confocal Imaging

Cells were grown to 80% confluency in Lab-Tek chamber slides (ThermoFisher, 177399), fixed in 4% paraformaldehyde for 10 min, and then washed with PBS three times for 5 min. Cells were permeabilized in 0.1% Triton X-100 and then blocked in 1% BSA/PBS for 1 h. Cells were incubated in E-cadherin antibody (1:200 in 0.1% BSA in PBS, Cell Signaling, 3195) overnight at 4 °C. Cells were washed in 0.05% Tween in PBS (3 × 5 min). Secondary detection was performed with goat anti-rabbit Alexa Fluor 488 (1:500, ThermoFisher, A-21206) for 1 h at room temperature. Cells were washed again with 0.05% Tween in PBS (3 × 5 min), and DAPI (NucBlue, ThermoFisher, R37606) was added during the last PBS wash (1 drop per mL). The chambers were removed, along with excess liquid. ProLong Glass Antifade Mountant (ThermoFisher, P36984) was added to the slide, coverslips were placed on top, and then the slide was allowed to dry overnight at 4 °C. Images were collected with a C2 Nikon confocal microscope using 0.5–1.0 μm steps and compiled using the maximum intensity function to compress the z-stacks. All experimental images were adjusted for brightness and color balance in the same way.

## 3. Results

### 3.1. GBA Genomic and Proteomic Alterations Are Common in Human Cancers

**Figure 1:**
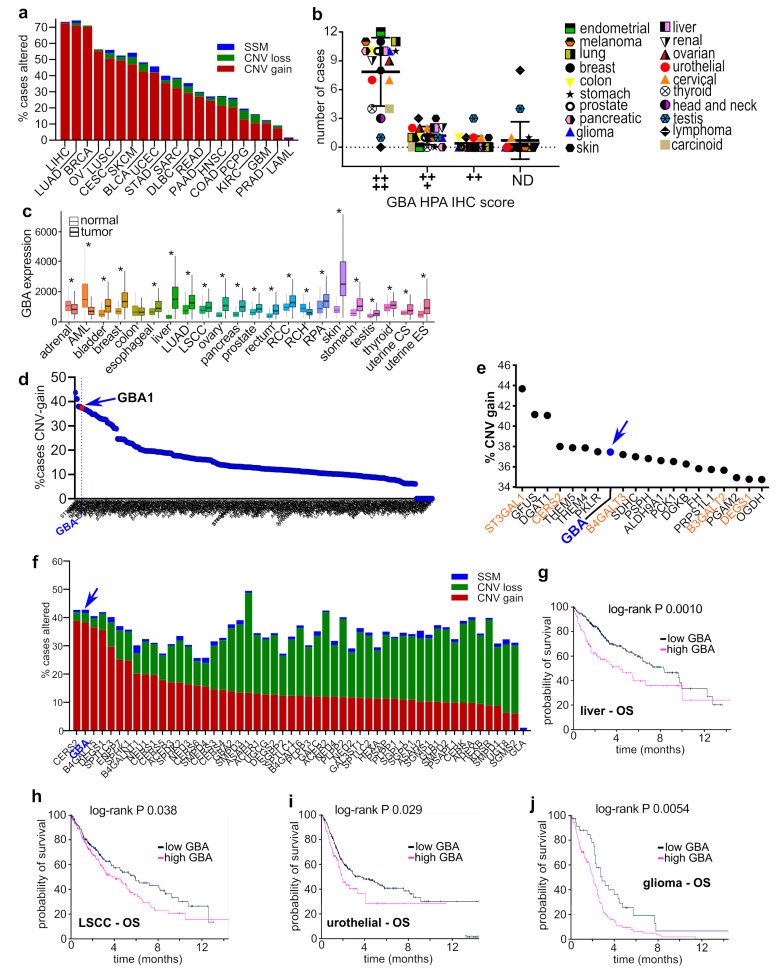
**GBA copy number amplifications and increased expression are highly prevalent in human cancers, and are associated with poor outcomes**. (**a**) TCGA data were queried for genomic alterations in GBA (ENSG00000177628) and plotted as the percentage of cases altered in the database by CNV gain alterations (red segments), CNV loss alterations (green segments), and simple somatic mutations (SSM, blue segments). (**b**) GBA staining profiles of cancers in the Human Protein Atlas. Plot shows the number of cases with high, medium, low, or nondetectable staining of GBA. (**c**) GBA expression was examined using the TNMplot database. The Mann–Whitney U test determined significant differences between normal and tumor tissue, marked with an asterisk (*). (**d**) Pan-cancer TCGA data were analyzed for CNV gain alterations in genes of sphingolipid, lacto, neolacto, ganglio, isoglobo, and globo glycosphingolipids, fatty acid synthesis, elongation, and degradation, citrate and pentose phosphate cycles, pyruvate, glycolysis and gluconeogenesis, fructose, mannose, galactose, glycine, serine, threonine, and glycerolipid metabolic pathways. (**e**) Top 20 genes with CNV gain alterations in (**d**). (**f**) TCGA pan-cancer data were examined for genomic alterations in core sphingolipid metabolism genes (Kegg; hsa00600) and plotted as the percentage of subjects with the indicated genetic lesions within the cohort. (**g**–**j**) Kaplan–Meier plots generated with the Human Protein Atlas online tool for GBA and the indicated cancers and outcome measures.

To evaluate the prevalence of GBA alterations in human cancers, we examined genomic data available from The Cancer Genome Atlas (TCGA). As shown in Figure 1a, copy number variant (CNV) gain mutations were observed in a high percentage of tumors from patients with cancers of the liver (72.6%), lung (LUAD, 72.6%; LUSC, 50.7%), breast (70.1%), ovaries (55.1%), cervix (49.5%), skin (47.2%), bladder (42.7%), uterus (41.8%), and others. Importantly, these data also revealed that simple somatic mutations and CNV loss of GBA are uncommon (Figure 1a). Given that there is a correlation between CNV gain mutations and differential gene expression in cancer [28], these findings suggest that GBA expression is likely increased in these cancers. To examine this, we evaluated immunohistochemistry staining data from the Human Protein Atlas pathology project to assess GBA protein levels in tumors [29]. GBA staining was very high in 15 of 17 cancers evaluated, with only lymphoma and testicular cancer not showing very high GBA staining (Figure 1b). Lastly, the relative expression levels of GBA in normal and tumor tissues were evaluated using the TNMPlot transcriptomic cancer database and analysis tool [30]. Relative to normal tissues, GBA expression levels were significantly elevated in the tumors of 20 out of 22 cancers (Figure 1c). In combination, these data indicate that GBA alterations on genomic, mRNA, and protein levels are prevalent characteristics in the tumors of patients with various types of cancer.

Given that metabolic alterations are a hallmark of human cancers, we sought to determine, relative to other metabolism genes, how common GBA somatic CNV gain alterations are across pan-cancer data. The Kegg database [31] was queried for genes in the core sphingolipid pathway (Kegg hsa00600), glycosphingolipid lacto and neolacto series (Kegg hsa00601), ganglio (Kegg hsa00604), isoglobo and globo series (Kegg hsa00603), fatty acid synthesis (Kegg hsa00061), elongation (Kegg hsa00062), and degradation (Kegg hsa00071), citrate (Kegg hsa00020) and pentose phosphate cycles (Kegg hsa00030), pyruvate (Kegg hsa00620), glycolysis and gluconeogenesis (Kegg has00010), fructose and mannose (Kegg hsa00051), galactose (Kegg hsa00052), glycine, serine and threonine metabolism (Kegg hsa00260), and glycerolipid (Kegg hsa00561) metabolic pathways. Pan-cancer genomic alterations were evaluated in TCGA data available through the Genomic Data Commons Data Portal [32]. As shown in Figure 1d, out of 524 genes included in the analysis, GBA alterations ranked eighth overall with 37.5% of patients in the pan-cancer analysis cohort (13,714 patients) having CNV gain mutations. Other sphingolipid pathway genes with CNV gain alterations also ranked in the top 20 including ST3GAL1 (first), CERS2 (fourth), B4GALT3 (ninth), B3GALT2 (17th), and DEGS1 (19th) (Figure 1e). GBA simple somatic mutations were observed in 1.15% of patients, and CNV loss mutations were observed in 3.11% of patients, suggesting that these are not enriched genetic lesions in tumors. Among sphingolipid genes in the core biosynthetic pathways that synthesize ceramide, GlcCer, and its precursors, GBA was the second highest altered gene in pan-cancer data (Figure 1f).

Lastly, associations between GBA transcript levels and outcomes were evaluated using the Human Protein Atlas survival analysis tool [29]. High levels of GBA transcript were significantly associated with decreased overall survival in liver (Figure 1g), lung squamous cell carcinoma (Figure 1h), urothelial cancer (Figure 1i), and glioma (Figure 1j). In combination, these results suggest that GBA amplifications and increased protein expression are common alterations in the tumors of patients with cancer and impact outcomes.

### 3.2. Depletion of GBA in HeLa and H1703 Cells Resulted in GlcCer Accumulation and Alters Their EMT/MET State

**Figure 2:**
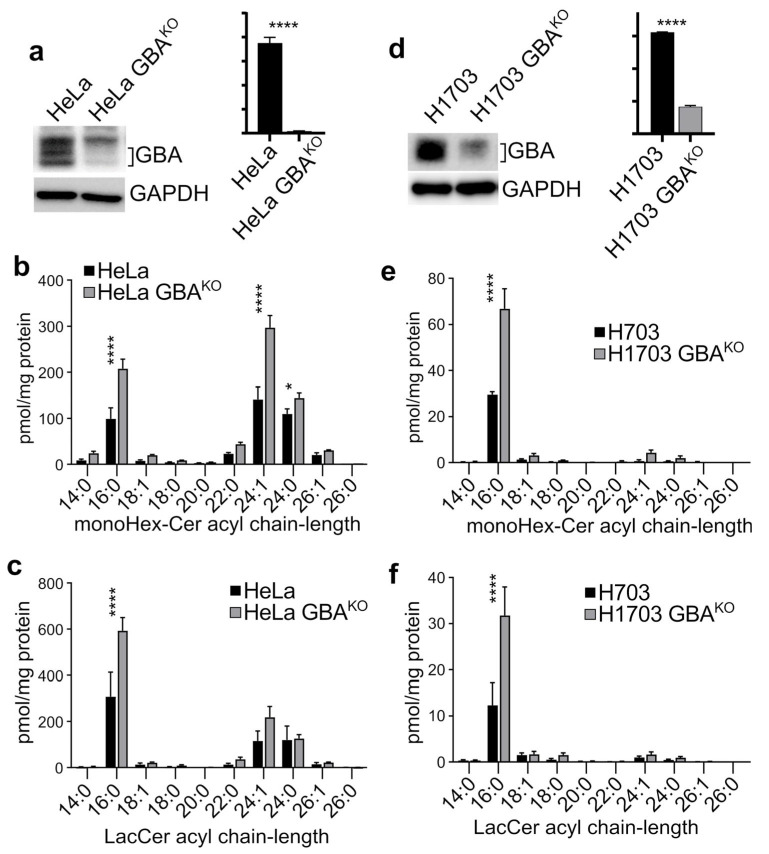
**Deletion of GBA resulted in the accumulation of GlcCer and LacCer in HeLa and H1703 cells.** (**a**,**d**) Immunoblots and densitometry analysis with the indicated antibodies of HeLa (**a**) and H1703 (**d**) cells in which GBA was deleted using CRISPR/Cas9; n = 3 each line. (**b**,**c**,**e**,**f**) Mass spectrometry analysis of the indicated lipids in HeLa/HeLa GBA^KO^ (**b**,**c**) and H1703/H1703GBA^KO^ (**e**,**f**) cells. Lipids were normalized per mg of protein input; n = 3 each line. Plots are bar graphs with the mean ± SEM; *t*-test analysis (Prism): ns, not significant; * *p* ≤ 0.05; **** *p* ≤ 0.0001. Blots were quantified with ImageJ.

Since GBA was elevated in many cancers, we asked what effect depleting it would have on various phenotypic properties associated with cancer malignancy. We used CRISPR/Cas9 to generate genetically depleted GBA^KO^ cell lines. GBA was targeted in the cervical SCC cell line HeLa and in the lung SCC cell line NCI-H1703. As shown in Figure 2a, GBA levels were significantly reduced in HeLa cells. Because GBA is the last step in the degradation pathway of glycosylated sphingolipids, a reduction in its expression levels should lead to the accumulation of GlcCer. We used mass spectrometry to measure the levels of GlcCer and its immediate derivative LacCer. As expected, GBA resulted in the significant accumulation of monohexosylceramides (monoHex-Cer), and specifically of monoHex-Cer species of 16:0, 24:1, and 24:0 fatty acyl chain length (Figure 2b). HeLa GBAKO cells also had significant accumulation of 16:0 LacCer (Figure 2c). In H1703 cells, CRISPR/Cas9 also resulted in a significant reduction in GBA levels (Figure 2d), as well as a significant accumulation of monoHex-Cer (Figure 2e) and LacCer (Figure 2f).

**Figure 3:**
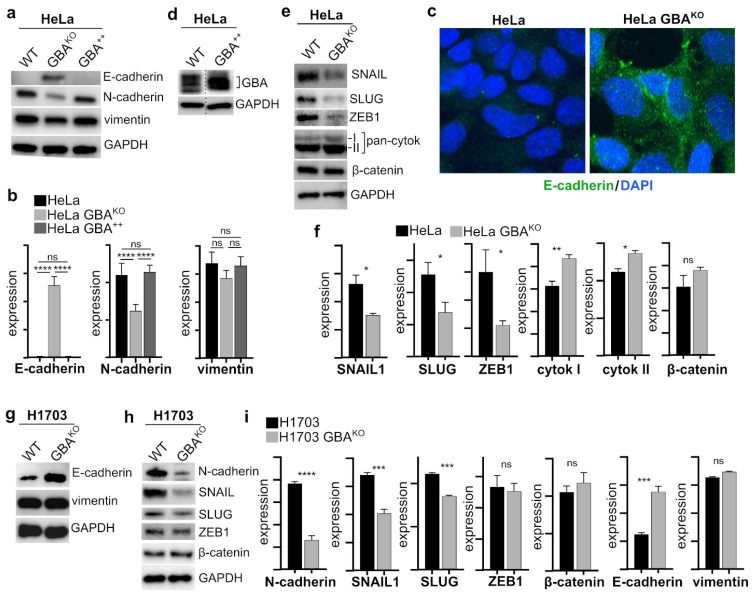
**HeLa GBA^KO^ and H1703 GBA^KO^ cells have significant reduced expression of EMT markers.** Immunoblots (**a**,**d**,**e**) and corresponding densitometry (**b**,**f**) analysis of the indicated proteins in HeLa (**a**,**d**,**e**), HeLa cells overexpressing GBA (**b**,**d**), and HeLa GBA^KO^ cells (**a**,**e**). All cell lines, n = 3. (**c**) Confocal images of HeLa and HeLa GBA^KO^ cells probed with the indicated antibodies. Nuclei were labeled with DAPI. (**g**–**i**) Immunoblots (**g**,**h**) and corresponding densitometry analysis (**i**) of the indicated proteins in H1703 and H1703 GBA^KO^ cells; n = 3 both lines. Plots are bar graphs with the mean ± SEM. In (**b**), statistical tests were performed with ANOVA with multiple comparisons (Prism); in (**f**,**i**), a *t*-test (Prism) was performed: ns, not significant; * *p* ≤ 0.05; ** *p* ≤ 0.01; *** *p* ≤ 0.005; **** *p* ≤ 0.0001. Blots were quantified with ImageJ.

Because GSLs are known modulators of RTKs that regulate EMT [33], the expression of established EMT markers was examined in GBA^KO^ cells. E-cadherin is a key element of adherens junctions in epithelial cells and is considered a key marker of cells that have epithelial-like characteristics [34]. Moreover, it is downregulated during EMT and in malignant epithelial cancers [33]. In HeLa cells, depletion of GBA resulted in a significant increase in E-cadherin (Figure 3a–c). However, further overexpression of GBA in HeLa cells (Figure 3d) did not significantly change E-cadherin levels (Figure 3b).

N-cadherin is often upregulated in cancer cells that have undergone EMT [33]. A decrease in E-cadherin and increase in N-cadherin expression, or a “cadherin switch”, is associated with increased malignancy and poor outcomes, and is characteristic of mesenchymal-like cells [34]. Consistently, in HeLa cells, GBA depletion resulted in a significant decrease in N-cadherin (Figure 3a,b), but there was no change in N-cadherin in HeLa cells overexpressing GBA (Figure 3a,b). Other important markers of EMT include proteins that are involved in the formation of intermediate filaments such as vimentin and cytokeratin, with vimentin expressed in mesenchymal-like cells and cytokeratin in epithelial-like cells [35]. In HeLa cells, GBA depletion resulted in a nonsignificant reduction in vimentin (Figure 3a,b), but a significant increase in cytokeratin (Figure 3e,f). GBA overexpression did not impact vimentin levels (Figure 3a,b).

The transcription factors SNAIL, SLUG, and ZEB1 are key regulators of EMT by modulating the expression levels of E-cadherin, N-cadherin, vimentin, and other proteins [34], and their increased expression is associated with poor outcomes and aggressive disease in many types of cancer [36,37,38,39,40]. Given that GBA depletion resulted in a significant increase in E-cadherin, a significant decrease in N-cadherin, and a small but nonsignificant decrease in vimentin, we examined the levels of the transcription factors SNAIL, SLUG, and ZEB1 in GBA^KO^ cells. In HeLa GBA^KO^, these were all significantly lower (Figure 3e,f). SNAIL interacts with and stabilizes β-catenin, an important regulator of cell adhesion and interacting partner of E-cadherin, but there were no significant changes in β-catenin in GBA^KO^ cells (Figure 3e,f). Together, these results show that reducing the expression of GBA in HeLa cells resulted in remodeling of EMT markers consistent with a reduced EMT phenotype, including decreased expression of key EMT-specific transcription factors, an N- to E-cadherin switch, and increased expression of cytokeratin.

To determine if the changes in EMT markers were not only specific to HeLa cells, we also examined the effect on EMT state in the SCC H1703 cell line. GBA-depleted H1703 cells also had significantly higher levels of E-cadherin (Figure 3g,i), as well as significantly lower N-cadherin, SNAIL, and SLUG (Figure 3h,i), but no significant change in ZEB1 (Figure 3h,i). These cells also had no significant changes in β-catenin (Figure 3h,i) or vimentin (Figure 3g,i). Therefore, these results show that reducing GBA expression in tumor-derived SCC cell lines results in MET phenotypic shift.

### 3.3. The GBA Inhibitor CβE Did Not Affect EMT Status

**Figure 4:**
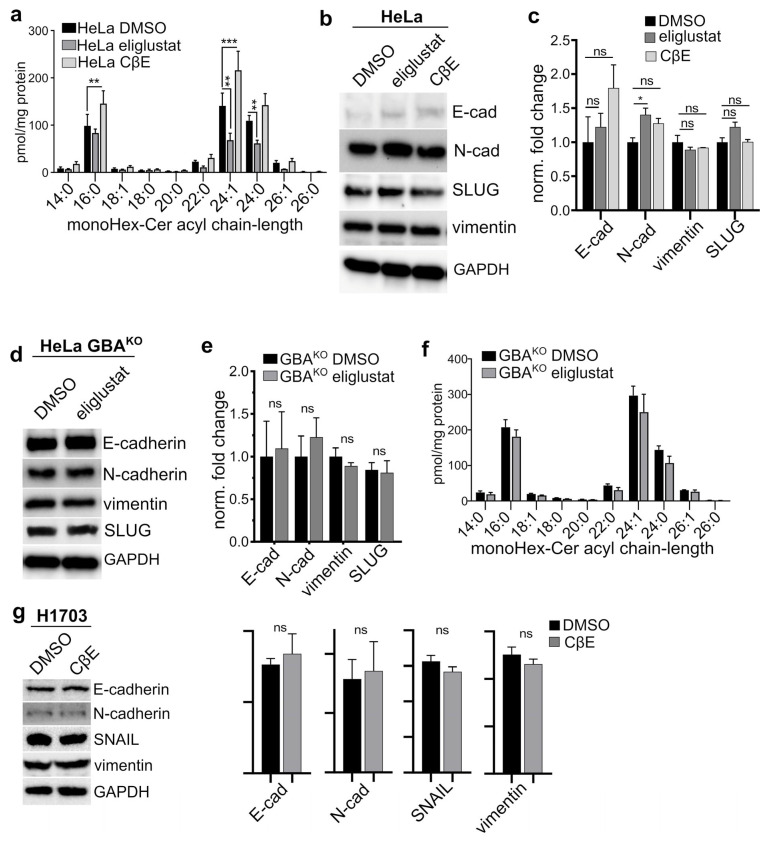
**Inhibition of GlcCer synthase or GlcCer degradation in HeLa, GlcCer synthase inhibition in HeLa GBA^KO^, or GlcCer degradation in H1703 did not affect EMT marker levels.** (**a**) MonoHex-Cer mass spectrometry analysis of HeLa cells treated with DMSO (0.01% *w*/*v*), 1 µM eliglustat tartrate, or 35 µM CβE for 15 days with biweekly medium changes; N = 3. (**b**,**c**) Immunoblots (**b**) and corresponding densitometry (**c**) analysis with the indicated antibodies of cells treated in panel (**a**). (**d**–**f**) Immunoblots (**d**), corresponding densitometry (**e**), and mass spectrometry (**f**) analysis of HeLa GBA^KO^ cells treated with DMSO (0.01% *w*/*v*) or 1 µM eliglustat tartrate for 15 days with biweekly medium changes. (**g**) Immunoblots and corresponding densitometry analysis of H1703 cells treated with treated with DMSO (0.01% *w*/*v*) or 35 µM CβE for 10 days with biweekly medium changes; N = 3 all cell lines. Plots are bar graphs with the mean ± SEM. In (**a**,**c**,**e**,**f**), statistical tests were performed with ANOVA and multiple comparisons; in (**g**) *t*-test (Prism) was performed: ns, not significant; * *p* ≤ 0.05; ** *p* ≤ 0.01; *** *p* ≤ 0.005. Blots were quantified with ImageJ.

Given that genetic depletion of GBA led to an apparent MET shift in both HeLa and H1703 cells, we next determined whether pharmacological modulation of GlcCer levels would have similar effects. In hepatocellular carcinoma-derived lines, the effect of GBA deletion was overcome with a GlcCer synthase inhibitor [22]. HeLa cells were treated with a vehicle, with the GlcCer synthase inhibitor eliglustat tartrate [41], or with the β-glycosidase inhibitor conduritol β-epoxide (CβE). To better mimic the prolonged effects of genetic deletion of GBA, HeLa cells were treated with DMSO (vehicle), 35 µM CβE, or 1 µM eliglustat for 2 weeks with biweekly medium changes. As expected, compared to controls, CβE-treated HeLa had a significant accumulation of monoHex-Cer (Figure 4a), and eliglustat-treated cells had significantly lower monoHex-Cer (Figure 4a). However, CβE treatment did not significantly alter the levels of E-cadherin (Figure 4b,c), N-cadherin, vimentin, or SLUG (Figure 4b,c). Eliglustat treatment resulted in a small but significant increase in N-cadherin, but had no effect on E-cadherin, vimentin, of SLUG (Figure 4b,c).

To assess if the effect on EMT marker expression in HeLa GBA^KO^ cells was due to accumulated GlcCer, we examined if inhibiting GlcCer synthase in HeLa GBA^KO^ cells would reverse this phenotype and make them more mesenchymal-like. HeLa GBA^KO^ cells were treated with 1 µM eliglustat tartrate for 2 weeks with biweekly medium changes. There were no significant changes in the levels of E-cadherin, N-cadherin, vimentin, or SLUG in these cells (Figure 4d,e). In addition, surprisingly, eliglustat did not significantly decrease monoHex-Cer levels (Figure 4f); we speculate that, in these cells, the accumulation of monoHex-Cer driven by reduced GBA expression counteracts the effect of GlcCer synthase inhibition. In H1703 cells, treatment with CβE did not significantly change the levels of E-cadherin, N-cadherin, vimentin, or SNAIL (Figure 4g). These results suggest that pharmacological modulation of GBA with CβE or of GlcCer synthase with eliglustat is insufficient to induce the MET phenotypic changes elicited by genetic depletion of GBA. In support of this, untargeted lipidomic experiments described below provide evidence that the effects of CβE and GBA genetic depletion on the cellular GSL lipidome are vastly different.

### 3.4. GBA Depletion Reduces the Migratory and Invasive Capacity of SCC Cells

**Figure 5:**
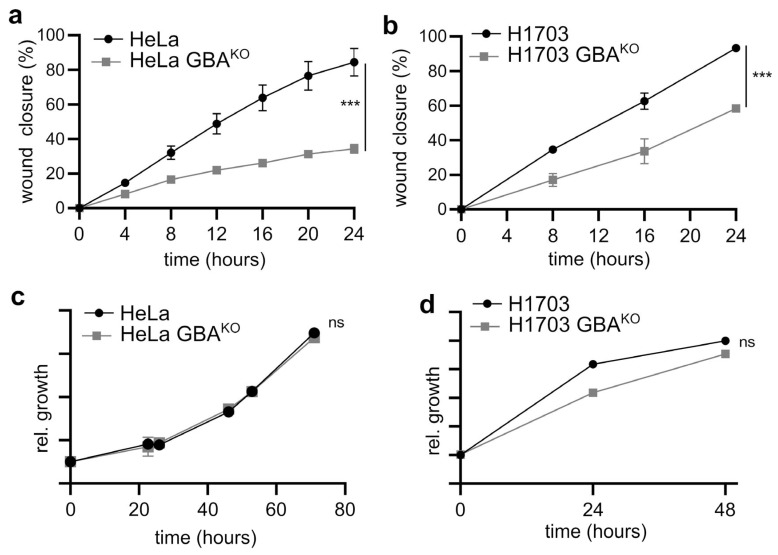

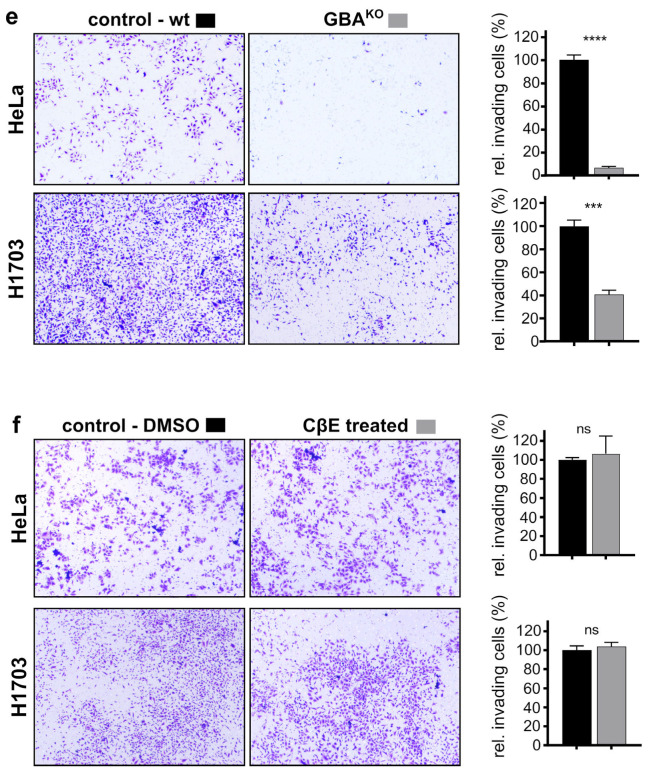
**GBA depletion reduced the migratory and invasive capacity of HeLa and H1703 cells, but CβE did not affect their invasive capacity**. (**a**,**b**) Cell migration in HeLa (**a**) and H1703 (**b**) was assayed after a linear segment of confluent cells was removed by scratching. Cells were grown at 37 °C, 5% CO_2_, and imaged at the indicated times; N = 3 for each line. Wound closure was plotted as the wound closure relative to that measured at t = 0 h. (**c**,**d**) The growth of HeLa/HeLa GBA^KO^ (**c**) and H1703/H1703 GBA^KO^ (**d**) cells was monitored in 96-well plates using WST-8 assays at the indicated times; N = 5 each line. In (**a**–**d**), graphs are scatter plots with the mean ± SEM. ANOVA (Prism): ns, not significant; *** *p* ≤ 0.005. (**e**,**f**) Representative images and corresponding quantification of the indicated cell lines that migrated through a Matrigel matrix and were stained with crystal violet. In (**f**), cells were treated with either DMSO (0.01% *w*/*v*) or 35 µM CβE for 10 days with biweekly medium changes. For all panels, cells were pre-starved for 2 h prior to seeding in the upper chamber of a transwell insert containing Matrigel. Cells were allowed to grow for 48 h at 37 °C and 5% CO_2_. The lower chamber contained medium supplemented with 10% FBS. Plots are bar graphs with the mean ± SEM; n = 3 for each line. *t*-Test analysis (Prism): *** *p* ≤ 0.005; **** *p* ≤ 0.0001.

Cadherins and intermediate filaments are elements of a complex cellular network of proteins that maintain epithelial tissues by stabilizing cell-to-cell contacts and maintaining adhesion [35]. Therefore, changes in the expression of E- and N-cadherin, as well as of intermediate filament proteins, are associated with a shift in the migratory and invasive capacity of cells [42]. Scratch assays and extracellular matrix invasion assays were performed to probe the migratory and invasive capacity of GBA^KO^ cells. As shown in Figure 5a,b, GBA-depleted HeLa and H1703 cells had significantly reduced migration rates in scratch assays. Importantly, in neither cell line did deletion of GBA significantly affect their growth rate (Figure 5c and Figure 5d, respectively), suggesting that the effect is migration- and not proliferation-dependent. In Matrigel invasion assays, GBA^KO^ HeLa and H1703 cells had significantly reduced capacity to cross the extracellular matrix (Figure 5e). However, extended treatments with the GBA inhibitor CβE did not significantly affect the invasive capacity of HeLa or H1703 cells (Figure 5f). These results support the findings that GBA^KO^ cells have the proteomic markers, migratory, and invasive phenotypic properties consistent with being MET shifted relative to cells expressing high levels of GBA.

### 3.5. GBA-Depleted SCC Cells Are Sensitized to Chemotherapeutic Agents

**Figure 6:**
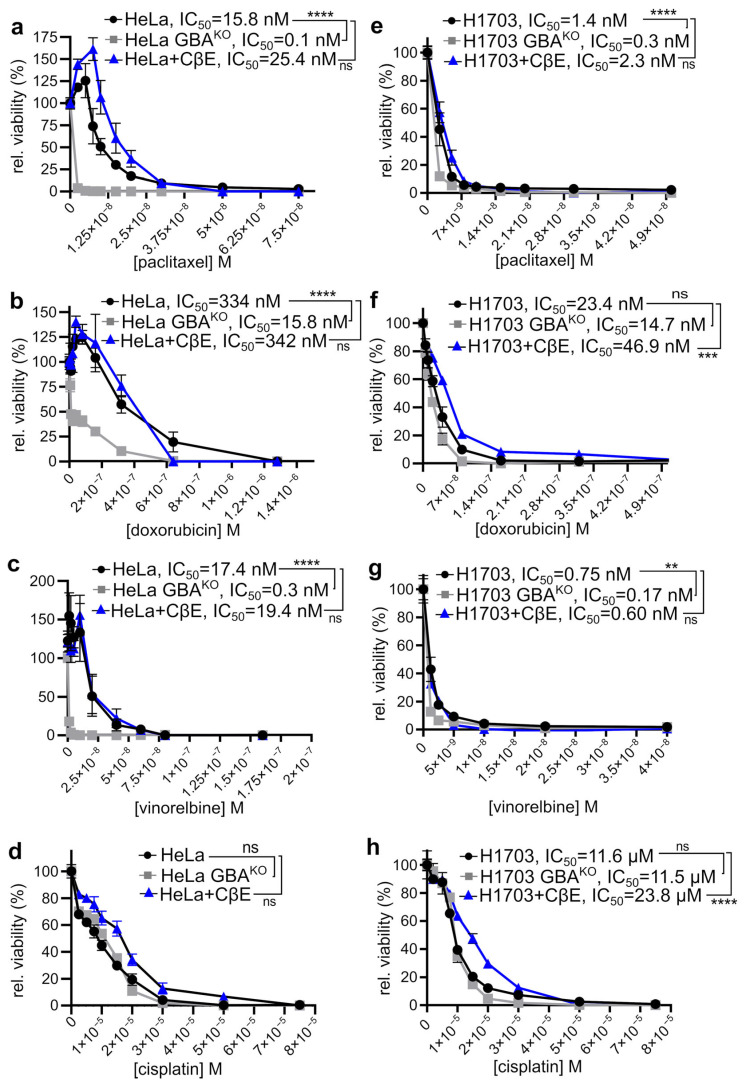
**GBA depletion increased the sensitivity of HeLa and H1703 to chemotherapeutic agents, but treatment with CβE did not**. (**a**–**h**) HeLa and HeLa GBA^KO^ (**a**–**d**), H1703 and H1703 GBA^KO^ (**e**–**h**), or HeLa or H1703 cells treated with 35 µM CβE for 15 days with biweekly medium changes were treated with the indicated drugs and concentrations for 72 h, and viability was assayed with WST-8. Cells were grown at 37 °C and 5% CO_2_. IC_50_ fitting and ANOVA were performed with Prism; ns, not significant; ** *p* ≤ 0.01; *** *p* ≤ 0.005; **** *p* ≤ 0.0001; n = 3 each line.

Chemoresistance is strongly associated with both increased GlcCer levels and EMT shift [7,43,44,45]. Therefore, given that deletion of GBA resulted in both accumulation of GlcCer and a phenotypic MET shift, we examined if there were accompanying changes in their sensitivity to various clinically relevant chemotherapeutic agents. Cells were exposed to drugs for 72 h and then cellular viability examined by monitoring the conversion of WST-8 to WST-8 formazan. Depletion of GBA in HeLa resulted in a significant decrease in viability when cells were treated with paclitaxel, doxorubicin, and vinorelbine (Figure 6a–c, respectively). Importantly, as compared to otherwise isogenic HeLa cells, the IC_50_ for HeLa GBA knockout cells was 158-fold lower for paclitaxel (Figure 6a), 21-fold lower for doxorubicin (Figure 6b), and 57-fold lower for vinorelbine (Figure 6c). GBA depletion did not significantly affect the sensitivity of HeLa cells to cisplatin (Figure 6d). Similar but less profound effects on cell viability were observed in H1703 cells. Compared to their wildtype parental cells, H1703 GBA^KO^ cells also had a significantly lower IC_50_ for paclitaxel (Figure 6e; 4.6-fold), doxorubicin (Figure 6f; 1.5-fold), and vinorelbine (Figure 6g; 4.4-fold). GBA depletion in H1703 also did not significantly affect their sensitivity to cisplatin (Figure 6h). Thus, GBA depletion in SCC cancer cell lines not only makes them less metastatic-like but also more sensitive to a subset of clinically relevant chemotherapeutics. For all drugs and both cell lines, cotreatment with CβE did not significantly increase their chemosensitivity (Figure 6a–h).

### 3.6. GBA Depletion Results in Broad Changes Receptor Tyrosine Kinase Activation

**Figure 7:**
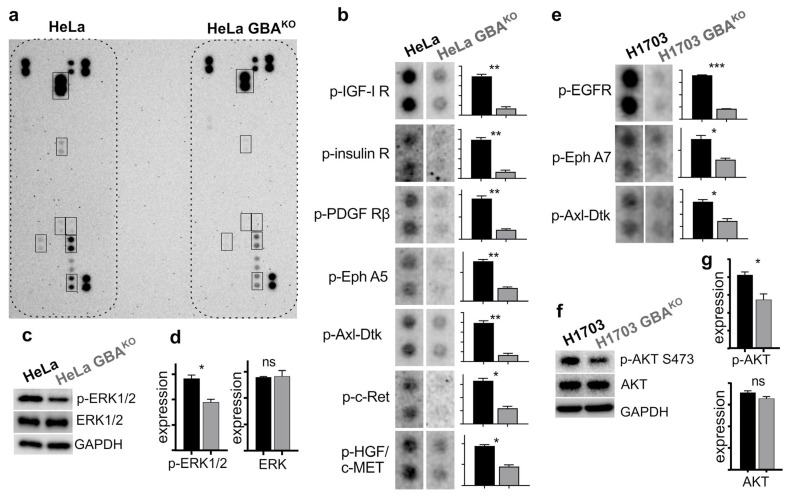
**GBA^KO^ cells have significantly decreased activation of RTKs. (a) Representative image of a Proteome Profiler Human RTK array.** For assays, cells were serum-starved for 3 h and then incubated with medium containing 10% FBS for 30 min. Arrays were developed and imaged simultaneously as shown. Spots outlined are shown in (**b**). (**b**) Spots for the indicated RTKs in (**a**) were quantified. (**c**,**d**) Cell extracts prepared for experiments in (**a**) were analyzed by immunoblotting (**c**) with the indicated antibodies and quantified by densitometry (**d**). Extracts were run in triplicate. *t*-Tests were performed with Prism. (**e**–**g**) H1703 and H1703 GBA^KO^ cells were treated as cells in (**a**), and then incubated with Profiler arrays, before quantifying selected spots (**e**). Extracts were analyzed by immunoblotting (**f**) and quantified by densitometry (**g**). Extracts were run in triplicate. All *t*-tests were performed with Prism 8, and dots were quantified by densitometry using ImageJ 1.53. ns, not significant; * *p* ≤ 0.05; ** *p* ≤ 0.01; *** *p* ≤ 0.0005.

EMT is a complex molecular phenotypic transformation that involves the activation and regulation of multiple cellular factors. Some of the key players in the induction of EMT are RTKs, which are upstream of transcription factors such as SNAIL and SLUG [46]. Therefore, the activity of RTKs can modulate changes in the expression profile of proteins involved in the maintenance of cell junctions and other processes involved in EMT. Furthermore, RTKs also play an important role in chemoresistance [47]. Because we observed that GBA depletion in HeLa and H1703 cells resulted in significant changes that suggested an MET shift, in addition to being more sensitive to some chemotherapy drugs, we conducted experiments to determine if there were any changes in their global RTK activation profile. We used phospho-RTK proteome profiler arrays (R&D Systems), which can detect 49 phosphorylated RTKs using cell lysates. Experiments used cell extracts from HeLa and H1703 GBA^KO^ cells that had been serum-starved for 3 h and then stimulated by incubating cells with medium containing 10% FBS for 30 min. FBS is rich in growth factors and hormones [48]. Input lysates were normalized, and profiler membranes were developed and imaged in parallel to avoid experimental artifacts. Extracts were also analyzed for downstream signal transduction relays protein kinase B/AKT or ERK1/2. Relative to their otherwise isogenic wildtype counterparts, HeLa GBA^KO^ had significantly reduced phosphorylated insulin and insulin-like growth factor 1 receptor (IGF-I R), platelet-derived growth factor receptor-β (PDGF-R β), ephrin A5 receptor (Eph A5), Axl family receptor (Axl-Dtk), c-Ret, and hepatocyte growth factor receptor (HGF/c-MET) (Figure 7a,b). HeLa GBA^KO^ input lysates also had significantly reduced levels of ERK1/2 (Figure 7c), but no changes in total levels of ERK1/2 (Figure 7c,d). In H1703 GBA^KO^ cells, there were significant reductions of phosphorylated epidermal growth factor receptor (EGFR), ephrin A7 receptor (Eph A7), and Axl-Dtk (Figure 7e). H1703 GBA^KO^ input lysates also had significantly reduced serine-473 phosphorylated protein kinase B/Akt (Figure 7f), but no changes in total levels of Akt (Figure 7f,g). Thus, GBA depletion reduced the activation of a number of RTKs, and of downstream targets such as AKT and ERK1/2.

### 3.7. GBA Depletion Remodels the Cellular GSL Profile

Since GBA depletion had a broad effect on RTK activation, we then hypothesized that GBA activity might affect the composition of the membrane, which in turn is integral for RTK function. The mammalian plasma membrane contains sphingolipids of many different chain lengths and modifications. These include sphingomyelins, ceramides, and glycosylated ceramides such as GlcCer. Glycosylated sphingolipids derived from GlcCer can be further modified to form highly complex and varied lipid derivatives that are commonly referred to as GSLs. These important cellular lipids have many roles at the plasma membrane, and GSLs such as gangliosides are known to modulate the activity and signaling of RTKs [2,5]. Therefore, given that GBA depletion resulted in significant changes in the phosphorylation of various RTKs, we wondered if there were broad effects on the cellular lipidomic and GSL profile of GBA^KO^ cells beyond GlcCer and LacCer alterations shown in Figure 2. To examine this, untargeted lipidomics was performed on HeLa and HeLa GBA^KO^ cells. In addition, given that treatment with CβE did not phenocopy the effects on EMT markers as seen by genetic depletion of GBA, untargeted analysis was also performed on HeLa cells that were treated with 35 µM CβE (HeLa + CβE) for 2 weeks. A total of 845 lipids were identified in this analysis.

**Figure 8:**
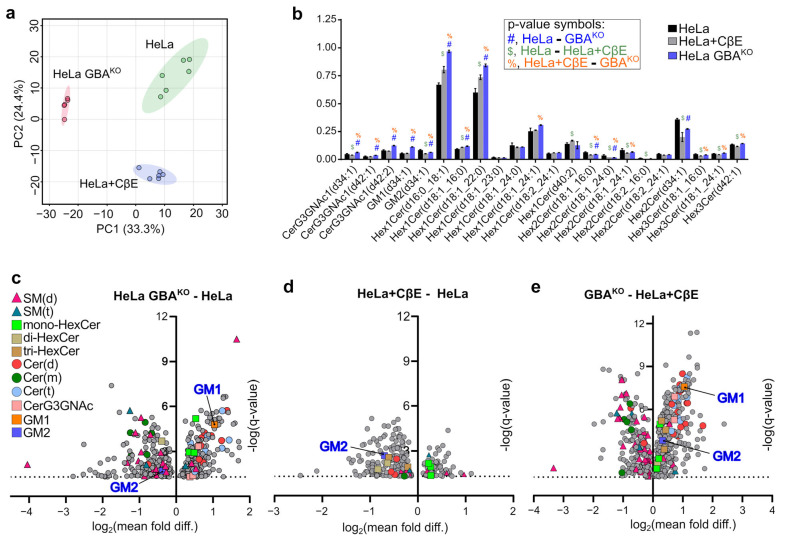
**HeLa, HeLa GBAKO, and HeLa cells treated with CβE have distinct lipidome, GSL, and sphingolipidome profiles.** (**a**) Untargeted lipidomics data were collected on HeLa, HeLa GBA^KO^, and HeLa cells treated with 35 µM CβE for 2 weeks (HeLa + CβE). HeLa and HeLa + CβE had n = 6 technical replicates, while HeLa GBA^KO^ had n = 5. A total of 845 lipid species were identified in data and were used to conduct a PCA analysis using MetaboAnalyst 5.0. A post hoc Tukey test with false discovery rate correction was used to compare the normalized annotated lipids every two groups, and the adjusted *p*-values of 0.05 and 0.1 were employed for significant differences. Hierarchical clusters were generated with R (version 4.2). (**b**) ANOVA corrected for multiple comparisons using FDR at Q = 5% (Prism) was used to identify statistically significant GSLs in untargeted data. Symbols used to indicate significant discoveries in pairwise comparisons are shown in the inset. (**c**) Volcano plots of indicated comparisons showing significant discoveries following ANOVA corrected for multiple comparisons using FDR at Q = 5%. Plots have Y-axes of −log_10_ (q-value), and X-axes are log_2_ of the ratio of means for the indicated comparisons: (**c**) HeLa GBA^KO^/HeLa; (**d**) HeLa + CβE/HeLa; (**e**) GBA^KO^/HeLa + CβE.

To visualize the structure of the untargeted lipidomics data and assess the similarities and differences among HeLa, HeLa GBA^KO^, and HeLa + CβE, principal component analysis (PCA) was performed. All 845 lipids identified were included in the analysis. As shown in Figure 8a, each group was distinctly clustered, and there were ample differences between groups. Importantly, there was a large variance in principal component 1 (PC1; 33.3%) between HeLa GBA^KO^ and both HeLa and HeLa + CβE. For PC2, there was less variance between HeLa GBA^KO^ and HeLa cells, but there was a large difference between HeLa GBA^KO^ and HeLa + CβE. PC2 contained 24.4% of the variance in the untargeted lipidomics dataset. These results suggest that the lipidome of HeLa GBA^KO^ cells is significantly different from that of HeLa and HeLa + CβE.

Given that GBA degrades GlcCer, which is one of the basic building blocks for most GSLs, we wondered if depletion of GBA might result in the differential accumulation of GSL species in HeLa and HeLa GBA^KO^ cells. Given that CβE targets GBA, but did not phenocopy the effects on EMT markers, we also examined differences between HeLa GBA^KO^ and HeLa + CβE GSL profiles, which may provide evidence to better understand these observed differences. HeLa GBA^KO^ cells had significantly higher levels of the ganglioside GM1 than HeLa, but lower levels of GM2 (Figure 8b). Compared to HeLa + CβE cells, GBA^KO^ cells had significantly higher levels of GM1 and GM2 (Figure 8b). Compared to untreated HeLa, HeLa + CβE cells did not have significantly different levels of GM1, but had lower GM2 (Figure 8b). Differences in GM1 and GM2 levels are more easily visualized in the volcano plots shown in Figure 8c–e.

There were also several notable differences in other GSLs. Consistent with data shown in Figure 2, HeLa GBA^KO^ cells had higher levels of various monoHexCers, as well as significantly higher levels of various N-acetylhexosyl-ceramides (Figure 8b). However, surprisingly, not all GSLs accumulated in HeLa GBA^KO^ cells, as they also had significantly lower levels of various GSLs including mono- and di-hexosylceramides (Figure 8b). This analysis also revealed that many GSLs were significantly different between HeLa GBA^KO^ and HeLa + CβE (Figure 8b), suggesting that the effect of CβE on the cellular GSL lipidome does not mimic that of GBA depletion. In combination, these results suggest that GBA depletion results in complex alterations of the cellular GSL lipidome, far beyond the expected effects on GlcCer accumulation.

To further visualize the complexity of the cellular sphingolipidome alterations resulting from GBA depletion, we generated volcano plots and highlighted sphingolipids by class. For this analysis, all identified lipids were compared using ANOVA with multiple comparisons and *p*-values adjusted using FDR and Q = 5%. Only discoveries are plotted in Figure 8c–e. As shown in Figure 8c, there was a broad and complex remodeling of the sphingolipidome in HeLa GBA^KO^ cells relative to HeLa, including significant changes in various sphingomyelins, ceramides, and GSLs. In comparison, HeLa + CβE had fewer significant sphingolipid alterations, suggesting that GBA depletion and CβE treatment have very different effects on the cellular sphingolipidome (Figure 8e).

## 4. Discussion

GBA is the only lysosomal enzyme known to catalyze the last step in the degradation of all GSLs, which are involved in the etiology, progression, chemoresistance, and responses to therapy in cancer. Here, we show that copy number amplifications and increased expression of GBA are highly enriched genetic lesions in human cancers. Moreover, relative to other genes within pathways associated with cancer hallmark metabolic alterations, GBA copy number amplifications rank among the highest. We also show that deleting GBA in two SCC-derived cell lines had significant effects on phenotypic properties associated with cancer malignancy and poor outcomes. Therefore, GBA should be considered a potentially important cancer therapeutic target.

### 4.1. Differential Effect of GBA Depletion in Various Cancer Cell Lines

It was previously shown that partial deletion of GBA in hepatocellular carcinoma-derived cell lines led to an EMT shift, decreased growth and invasive capacity, and increased metastatic potential [22]. However, it was also shown that inhibition of GlcCer synthase in the GBA^KO^ hepatocellular carcinoma-derived cells with 1-phenyl-2-decanoylamino-3-morpholino-1-propanol or miglustat reversed the effect on migration and invasion, and decreased N-cadherin and SLUG expression [22], thus suggesting the possibility that, in liver cancer-derived cells, GlcCer synthesis may be one of the main drivers of the phenotypic effects resulting from GBA deletion. In contrast, in our study, depleting GBA in SCC-derived cell lines had the opposite effect as it induced MET, decreased migratory and invasive capacity, and did not affect growth rate. Furthermore, we found that inhibiting GlcCer synthesis in GBA^KO^ cells with eliglustat did not reverse the effect on EMT markers, suggesting that the mechanism driving EMT-to-MET shift in these lines is primarily driven by GBA activity, and not by GlcCer synthesis. In ovarian cancer cell lines, GBA depletion using siRNA led to changes more consistent with our results, including decreasing their chemoresistance and the activity of the Axl RTK pathway [24]. We suggest that the contrasting effects of GBA deletion, particularly those in hepatocellular carcinoma, are likely due to differences in the molecular alterations of sphingolipids that take place during transformation in these histologically different tumor-derived cell lines. This is supported by our previous data showing that hepatocellular tumors have significantly decreased GlcCer and LacCer [8], opposite of what we observed in SCC tumors and other tumor histologies where they are increased [8,9]. On the basis of the difference in phenotypic sensitivity of GBA^KO^ hepatocellular carcinoma and SCC cells to GlcCer synthase inhibition, we speculate that malignancy associated with alterations in sphingolipid metabolism are driven by the activity of GlcCer synthase in hepatocellular carcinoma, and by GBA activity in SCC and ovarian cancer-derived cells. Further experiments may be needed with cell lines derived from other histologies to assess those in which GBA inhibition may be a viable target for cancer therapy.

### 4.2. Role of GBA on Chemoresistance

Increased GlcCer levels are associated with intrinsic and acquired MDR [7,49]. However, although GBA depletion increased GlcCer in HeLa and H1703 cells, it sensitized them to paclitaxel, vinorelbine, and doxorubicin. However, it did not impact cisplatin resistance, which was observed in ovarian cancer cells where GBA was downregulated with siRNAs [24], suggesting that the GBA effect on chemoresistance may be histologically specific. This is consistent with the observation that, in gastric cancer cells, treatment with the antimetabolite 5-FU led to increased expression of GBA [23]. Regardless, data presented here and those previously published [23,24] highlight that the relationship between GlcCer levels and chemoresistance may also depend, at least in part, on whether cells have a high dependence on GBA activity to maintain EMT. We propose this as GBA depletion driving GlcCer accumulation via a defect in the endo-lysosomal compartment, and not by increased GlcCer synthesis flux from the ER–Golgi biogenesis pathway. Therefore, GlcCer accumulation in GBA^KO^ cells does not require the activity of transporters such as p-glycoprotein, which drive MDR in this context [49,50,51,52,53]. Rather, in GBA-depleted cells, the increased sensitivity to cytotoxic agents is likely a consequence of the EMT-to-MET shift. This is consistent with the established observations that the EMT phenotype is considered a major determinant in MDR [45,54,55]. However, given that RTK activation can also regulate chemoresistance [47], it is also possible that the observed reduction in RTK activation in HeLa GBA^KO^ cells also plays a role in their increased chemosensitivity. Regardless, these results suggest that, in some cancers, concomitantly inhibiting GBA and GlcCer synthase could be a potentially viable therapeutic option to significantly decrease MDR. Furthermore, given that a large percentage of tumors of patients with cancer have alterations in GBA (see Figure 1a,b), there may be a sizable population that could benefit from this approach. Unfortunately, there are few GBA inhibitors, and the best-characterized one, CβE, even under extended treatments, did not phenocopy the effects of GBA depletion on EMT or the shift in the balance of gangliosides GM1 and GM2. This may be due in part to CβE inhibiting many other β-glycosidases [56], which may prevent the accumulation of specific GSLs that mediate this effect. This observation may also explain why GSL lipidome alterations in HeLa + CβE cells were so vastly different than those of HeLa GBA^KO^ cells. Unfortunately, these results strongly suggest that targeting GBA in cancer may require the development of second-generation GBA inhibitors that are more specific and phenocopy the effects of GBA depletion.

### 4.3. Link among GBA Activity, RTKs, and EMT

Although it has been shown that alterations in the activity of GBA result in changes in the processes that regulate EMT [22] or activation of the Axl RTK pathway [24], a mechanism that explains why GBA depletion results in these changes has not emerged. In this report, we provide evidence that GBA depletion in two SCC cell lines results in changes in the balance of GSLs and the gangliosides GM1 and GM2, which are known regulators of RTKs [2,25,57,58] involved in the regulation of EMT, including PDGFR, the insulin receptor, c-MET, and EGFR [2,25,57]. Therefore, we propose that the broad alterations in the activation of RTKs shown in Figure 7 are the result of GBA-depletion induced changes to the GSL balance of the plasma membrane, specifically gangliosides GM1 and GM2. This hypothesis is consistent with the effects of GBA activity deficiencies in Gaucher’s disease, which result in the accumulation of many different lipid species including sphingolipids [16,21,59], with previous studies showing that EMT in various cancer cell lines is accompanied by alterations in ceramide metabolism [60], and with the results of our untargeted analysis revealing broad lipid metabolism alterations in HeLa GBA^KO^ cells. Previously published work also contains indirect evidence for this hypothesis. It was shown that depletion of GBA in hepatocellular carcinoma cell lines shifts these cells toward EMT, and it was shown that this effect was mediated in part by activation of the Wnt pathway via increased phosphorylation of LRP6 [22]. Although this was not investigated, there is strong evidence that LRP6 can be cross-activated by several RTKs [61], suggesting the possibility that the changes observed in GBA-depleted hepatocellular carcinoma-derived cells may also be due in part by alterations in RTK activity. More direct evidence supporting our hypothesis was shown in ovarian cancer-derived cell lines where silencing of GBA with siRNA led to significantly decreased activation of the Axl receptor and Ser-473 phosphorylation of AKT [24]. In addition, in a Gaucher cell model induced by decreasing GBA activity, preliminary results showed lower Ser-473 AKT phosphorylation upon induction with an RTK agonist [21]. Consistently, in this report, we observed decreased phosphorylation of Axl/DTK and p-AKT in H1703 GBA^KO^ cells, and of ERK1/2 in HeLa GBA^KO^ cells. Moreover, we showed that GBA^KO^ cells had significant alterations in the activation of other RTKs involved in the regulation of EMT including the insulin and insulin-like growth factor receptors [62,63], PDGFR [64], Axl-Dtk [65], HGF/c-MET [66], and EGFR [67]. Results showing altered insulin receptor phosphorylation are also consistent with previous well-established observations that gangliosides can decrease its activation [68]. Therefore, we speculate that the MET shift observed in HeLa and H1703 GBA^KO^ cells is likely due to a remodeling of the plasma membrane GSL lipidome, leading to changes in RTK activity. Consequently, there is a reduction in the activation of transcription factors such as SNAIL and SLUG that repress the expression of epithelial-like proteins [69], allowing cells to produce proteins that sustain the MET phenotype, and diminishing their chemoresistance.

### 4.4. Limitations of the Study

A limitation of our study is that the CβE GBA inhibitor did not phenocopy the effects of genetically reducing GBA expression. We speculate that this is because CβE is not fully effective at blocking all GBA functions, nor is it highly specific since it has been shown to inhibit other β-glycosidases [56]. Therefore, it may be important for future studies to develop better inhibitors that phenocopy the broad effects induced by genetic depletion. Another limitation is that untargeted lipidomics examined gross changes in the lipid profiles of GBA^KO^ cells. While this is powerful in that it provides us with a global snapshot, it does not provide spatial resolution of all alterations. Therefore, whether these changes indeed affect the plasma membrane composition, lipid raft structures, and the endosomal or lysosomal compartments cannot be determined from these data.

## 5. Conclusions

In combination, results in this study suggest that GBA is so highly enriched in human cancer tumors because its activity may be required to fine tune the plasma membrane GSL content that is needed to sustain phenotypic characteristics of malignant disease, including EMT, chemoresistance, and invasive capacity. Therefore, although much attention has been focused on decreasing GlcCer levels to reduce chemoresistance in cancer, given the effects that GBA depletion had on multiple phenotypic measures of cancer malignancy, it may be prudent to consider examining GBA inhibition as an adjuvant to GlcCer synthase inhibition to overcome chemoresistance in SCC and other cancers.

## Data Availability

At this time, primary data are not publicly available.
